# Comparative Genomic Analysis of *Colletotrichum lini* Strains with Different Virulence on Flax

**DOI:** 10.3390/jof10010032

**Published:** 2023-12-31

**Authors:** Ekaterina M. Dvorianinova, Elizaveta A. Sigova, Timur D. Mollaev, Tatiana A. Rozhmina, Ludmila P. Kudryavtseva, Roman O. Novakovskiy, Anastasia A. Turba, Daiana A. Zhernova, Elena V. Borkhert, Elena N. Pushkova, Nataliya V. Melnikova, Alexey A. Dmitriev

**Affiliations:** 1 Engelhardt Institute of Molecular Biology, Russian Academy of Sciences, Moscow 119991, Russia; sigova.ea@phystech.edu (E.A.S.); mollaev_t@mail.ru (T.D.M.); 0legovich46@mail.ru (R.O.N.); anastas.turba@gmail.com (A.A.T.); zhernova.d@ya.ru (D.A.Z.); sashai@inbox.ru (E.V.B.); pushkova18@gmail.com (E.N.P.); mnv-4529264@yandex.ru (N.V.M.); 2 Moscow Institute of Physics and Technology, Moscow 141701, Russia; 3 Agrarian and Technological Institute, Peoples Friendship University of Russia (RUDN University), Moscow 117198, Russia; 4 Federal Research Center for Bast Fiber Crops, Torzhok 172002, Russia; tatyana_rozhmina@mail.ru (T.A.R.); lpkudryavtseva@icloud.com (L.P.K.); 5 Faculty of Biology, Lomonosov Moscow State University, Moscow 119234, Russia

**Keywords:** *Colletotrichum lini*, anthracnose, flax pathogen, virulence, nanopore sequencing, de novo genome assembly

## Abstract

*Colletotrichum lini* is a flax fungal pathogen. The genus comprises differently virulent strains, leading to significant yield losses. However, there were no attempts to investigate the molecular mechanisms of *C. lini* pathogenicity from high-quality genome assemblies until this study. In this work, we sequenced the genomes of three *C. lini* strains of high (#390-1), medium (#757), and low (#771) virulence. We obtained more than 100× genome coverage with Oxford Nanopore Technologies reads (N50 = 12.1, 6.1, 5.0 kb) and more than 50× genome coverage with Illumina data (150 + 150 bp). Several assembly strategies were tested. The final assemblies were obtained using the Canu–Racon ×2–Medaka–Polca scheme. The assembled genomes had a size of 54.0–55.3 Mb, 26–32 contigs, N50 values > 5 Mb, and BUSCO completeness > 96%. A comparative genomic analysis showed high similarity among mitochondrial and nuclear genomes. However, a rearrangement event and the loss of a 0.7 Mb contig were revealed. After genome annotation with Funannotate, secreting proteins were selected using SignalP, and candidate effectors were predicted among them using EffectorP. The analysis of the InterPro annotations of predicted effectors revealed unique protein categories in each strain. The assembled genomes and the conducted comparative analysis extend the knowledge of the genetic diversity of *C. lini* and form the basis for establishing the molecular mechanisms of its pathogenicity.

## 1. Introduction

The most important aim of modern agriculture is to meet the food and raw material demand of the growing human population. However, yield volumes of cultivated plants depend on numerous factors [1,2]. Fungal diseases of plants are often the primary cause of crop losses [3]. Thus, the susceptibility to various diseases can become a threat to the harvest and economic profits. The genus *Colletotrichum* is pathogenic to different plant species and often demonstrates severe virulence [4,5,6]. *Colletotrichum lini* is the causative agent of flax anthracnose [7]. The pathogen can reside in untreated flax seeds and starts the infection process in seedlings and mature plants [7]. The mature plant infected with *C. lini* shows stem canker and leaf spotting. For the plant seedlings, the infection can become fatal [8]. Thus, the production of two main products—flax oil and fiber—can be affected by harvest failure. In light of this fact, multifaceted studies on the fungus are highly important for anthracnose management.

Pathogenic *Colletotrichum* species have three main lifestyles [9]—biotrophic (hemibiotrophic), necrotrophic, and quiescent lifestyles. The genus *Colletotrichum* generally lacks true biotrophic species [9]. Its representatives are usually considered hemibiotrophic, as they establish the necrotrophic stage after the biotrophic stage [10]. Biotrophic fungi suppress the defense mechanisms of the host and mask the hyphae to obtain nutrients from the host [11]. This stage can be indispensable for further establishment of infection and the death of host cells [10]. The necrotrophic stage implies the secretion of fungal toxins and enzymes to absorb nutrients from the dead host cells [11]. The quiescent stage is the period of dormancy of a fungus until a signal from the surrounding media is detected [9]. Then, the fungus can complete its disease cycle [9]. Along with pathogenic species, endophytic *Colletotrichum* strains also occur [12,13]. Endophytes live in plant tissues and receive the nutrition from a plant without causing disease symptoms. Both *Colletotrichum* endophytes and pathogens produce metabolites with useful bioactivities [14,15].

*Colletotrichum* representatives are attributed to species complexes according to intra-specific and interspecific differences in phenotype and genotype [11]. To discriminate between fungal species, genetic barcodes were applied, e.g., GAPDH, HIS3, APN2, MAT1-2-1, GAP2-IGS, ACT, CHS-1, nrITS, and TUB2 [16]. However, there is no single universal barcode for all *Colletotrichum* species, as they demonstrate different efficiencies in various species complexes [17,18]. Thus, an ITS-based approach coupled with molecular characterization was applied for *Colletotrichum* isolates from strawberry tissues. However, the authors observed no association with geographic origin, presence of symptoms, plant species, or parts. In addition, the ITS marker failed to provide enough resolution for differentiation between *C. gloeosporioides* isolates [18]. Therefore, multi-locus analysis can be used for reliable results [16]. Liu et al. constructed a genome tree for 94 *Colletotrichum* species. The analysis of 1893 single-copy orthologs allowed allocation of the taken species to a range of species complexes [19].

Nevertheless, most comprehensive information can be extracted from full genomic sequences of the *Colletotrichum* species [20]. Comparative genomic analysis assists in studying the origins of pathogenicity and virulence [21]. Thus, *Colletotrichum* species possess a suite of potential pathogenicity genes, including effectors and CAZymes [22]. The comparison between the gene repertoire of fungal species can shed light on the difference in virulence degree of fungal isolates. Meanwhile, horizontal gene transfer events can play an important role in the evolution of pathogenicity. For instance, in *C. musae*, the analysis of minichromosome sequences revealed a set of genes that can undergo horizontal gene transfer [23].

In this study, we obtained the annotated genomes of three *C. lini* strains of different virulence. The comparative analysis between the obtained assemblies revealed a difference in effector gene content, a chromosome rearrangement, and the absence of a possible pathogenicity chromosome in the genome of the moderately virulent strain. The obtained data are a valuable source of information on the pathogenicity determinants of the flax anthracnose pathogen. Further in-depth research on *C. lini* genomes will suggest possible solutions to breeding anthracnose-resistant flax varieties.

## 2. Materials and Methods

### 2.1. Fungal Material

Fungal strains were provided as pure cultures by the Institute for Flax (Torzhok, Russia). Mycelium was provided in test tubes with potato dextrose agar. The following *C. lini* strains were used: highly virulent strain #390-1, moderately virulent strain #757, and lowly virulent strain #771. The virulence of the three strains was assessed by infecting two flax varieties: the resistant variety Leona and the susceptible variety Punjab. Plants were sprayed with a spore suspension (150–300 spores per cm^3^), and the degree of virulence was estimated on the 8–9th day after infection. If up to 30% of plants showed the symptoms of anthracnose, the virulence was considered low; 31–50%—medium; 51% and more—high.

### 2.2. DNA Extraction and Purification

DNA extraction was performed according to the previously developed protocol [24,25]. The DNA was used for library preparation for both the Oxford Nanopore Technologies (ONT) and Illumina platforms. The quality and quantity of the extracted DNA were evaluated with spectrophotometry (NanoDrop 2000C, Thermo Fisher Scientific, Waltham, MA, USA) and fluorometry (Qubit 4.0, Thermo Fisher Scientific, Waltham, MA, USA).

### 2.3. DNA Library Preparation and Sequencing on the Oxford Nanopore Technologies and Illumina Platforms

To prepare DNA libraries for sequencing on the ONT platform, the SQK-LSK109 Ligation Sequencing Kit (ONT, Oxford, UK) was used. Sequencing was performed on a PromethION instrument with the R9.4.1 flow cell (ONT, Oxford, UK).

Illumina libraries were sequenced on a NovaSeq 6000 (Illumina, San Diego, CA, USA) instrument (150 + 150 bp).

### 2.4. Genome Assembly

The obtained ONT reads were basecalled using Guppy 6.0.1 and the dna_r9.4.1_450bps_sup.cfg config file with quality filtration threshold min_qscore = 10. Porechop 0.2.4 was used for removing adapters (https://github.com/rrwick/Porechop, accessed on 14 September 2023). The obtained short Illumina reads were processed using Cutadapt 2.8 (adapters removal: -a AGATCGGAAGAG -A AGATCGGAAGAG) [26] and Trimmomatic 0.39 (trimming by quality: TRAILING:30, filtration by length: MINLEN:50) [27]. For the highly virulent strain #390-1, draft assemblies were performed using two types of assemblers. For assembling a genome solely from ONT reads, the following assemblers were used: Canu 2.2 (-nanopore-raw; -minInputCoverage = 5; -stopOnLowCoverage = 5; -genomeSize = 50 m) [28], Flye 2.8.1 (-genome-size 50,000,000) [29], and Goldrush 1.0.3 (G = 5e7) [30]. For a hybrid assembly from both ONT and Illumina reads, the following tools were used: Haslr 0.8a1 (-g 50 m, -x nanopore) [31], Masurca 4.1.0 (GRID_BATCH_SIZE = 500,000,000) [32], Spades 3.15.5 [33], and Unicycler 0.5.0 (--mode bold) [34]. For the moderately virulent strain #757 and the lowly virulent strain #771, draft assemblies were produced by Canu 2.2 (-nanopore-raw; -minInputCoverage = 5; -stopOnLowCoverage = 5; -genomeSize = 50 m). To analyze the quality of the obtained assemblies, completeness and contiguity statistics were calculated using BUSCO 5.3.2 (glomerellales_odb10) and QUAST 5.0.2 [35,36]. The following reference genome was used for calculating QUAST reference-based statistics: *C. higginsianum* IMI 349063 (NCBI Genome, GCA_001672515.1).

The obtained draft assemblies of the three strains were polished with ONT reads using Racon 1.4.20 (two iterations) [37] and Medaka 1.5.0 (https://github.com/nanoporetech/medaka, accessed on 14 September 2023). Polca (Masurca 4.1.0) was used for polishing with Illumina reads [38]. If required, read alignment was produced with Minimap2 [39] prior to polishing.

### 2.5. Genome Analysis

For genome annotation, Funannotate v1.8.9 was used according to the basic assembly preparation protocol (https://funannotate.readthedocs.io/en/latest/, accessed on 14 September 2023) including cleaning up repetitive contigs, sorting the assembly by length, repeat masking, and gene prediction (https://github.com/nextgenusfs/funannotate, accessed on 14 September 2023). The received protein sequences were analyzed using KEGG (Kyoto Encyclopedia of Genes and Genomes) BlastKOALA (KEGG Orthology And Links Annotation, https://www.kegg.jp/blastkoala/, accessed on 14 September 2023) [40] and InterProScan 5.65-97.0 (https://github.com/ebi-pf-team/interproscan, accessed on 14 September 2023) [41]. The annotated genome assemblies were aligned to each other using LAST 1471 (https://gitlab.com/mcfrith/last, accessed on 14 September 2023). Tidk 0.2.31 was used for the identification of telomeric repeats and their visualization (https://github.com/tolkit/telomeric-identifier, accessed on 14 September 2023). RepeatMasker 4.1.5 was used for the identification of repeat content (https://www.repeatmasker.org/, accessed on 14 September 2023) [42]. SignalP-6.0 predicted the presence of signal peptides in protein sequences received after genome annotation (https://services.healthtech.dtu.dk/services/SignalP-6.0/, accessed on 14 September 2023) [43]. Protein sequences containing signal peptides were analyzed with EffectorP-3.0 to predict effector proteins [44]. Mega 11.0.13 was used for sequence alignments (https://www.megasoftware.net/, accessed on 14 September 2023) [45].

## 3. Results

### 3.1. Genome Assembly and Polishing

Three strains of *C. lini* with different virulence and close morphological traits (conidia characteristics, sporulation rate, growth rate, and mycelium color) were chosen for the study. For the highly virulent strain #390-1, we obtained 5.6 Gb of ONT data (average read Q ≥ 10) with an N50 of 12.1 kb and 10 million Illumina reads (150 + 150 bp). For the moderately virulent strain #757, we obtained 8.7 Gb of ONT data (average read Q ≥ 10) with an N50 of 6.1 kb and 23 million Illumina reads (150 + 150 bp). For the lowly virulent strain #771, we obtained 7.1 Gb of ONT data (average read Q ≥ 10) with an N50 of 5.0 kb and 25 million Illumina reads (150 + 150 bp).

**Figure 1:**
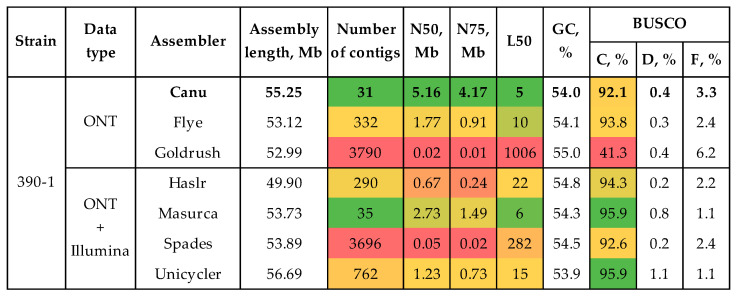
QUAST and BUSCO statistics of the highly virulent *C. lini* #390-1 draft genome assemblies. BUSCO: C—complete, D—duplicated, F—fragmented (the glomerellales_odb10 dataset). The used colors indicate estimations of the value quality: from bright green (best) to bright red (worst).

To test assembling and polishing algorithms, we used sequencing data of the highly virulent strain #390-1. We obtained three draft assemblies using only long ONT reads and four draft assemblies using both long ONT and short Illumina reads (Figure 1, Supplementary Table S1). The quality of the draft genome assemblies was analyzed in terms of completeness and contiguity using BUSCO and QUAST. For each assembly, QUAST statistics were evaluated without and with a reference genome of *C. higginsianum* IMI 349063 (NCBI Genome, GCA_001672515.1). The contiguity of the assemblies was judged by the number of contigs, N50, and L50. The assembly completeness was evaluated by the length and percentage of complete universal single-copy orthologs inherent to an analyzed species group.

The majority of tools produced assemblies with a BUSCO completeness of >90%, except Goldrush. The tool constructed an assembly with a completeness of 41.3%. The highest assembly completeness was achieved with hybrid assemblers (Masurca and Unicycler—BUSCO completeness of 95.9% in both cases). The average length of assemblies with a completeness of more than 90% was 53.8 Mb (49.9–56.7 Mb). The most contiguous assemblies were performed by Canu and Masurca. These assemblies had 31 and 35 contigs, respectively. The L50 values were also close—5 and 6, respectively. However, the N50 and N75 differed significantly between the two assemblies. The N50 values were 5.16 Mb and 2.73 Mb, and the N75 values were 4.17 Mb and 1.49 Mb, respectively. Thus, Canu assembled the genome of the best contiguity. Since the completeness of the draft assemblies can be improved by polishing, the assembly by Canu was regarded as optimal.

**Figure 2:**
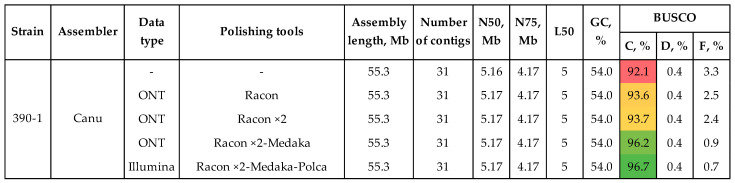
QUAST and BUSCO statistics of the polished genome assemblies of the highly virulent strain *C. lini* #390-1. BUSCO: C—complete, D—duplicated, F—fragmented (the glomerellales_odb10 dataset). The used colors indicate estimations of the value quality: from bright green (best) to bright red (worst).

The optimal genome assembly was polished according to the scheme that showed the best results in our previous studies [24,25]: polishing with long ONT reads—two rounds of Racon, one round of Medaka; polishing with high-precision Illumina reads—one round of Polca (Figure 2, Supplementary Table S2). Each round of polishing improved BUSCO completeness. The parameter reached 96.7% after Polca. Such features as the number of contigs, N50, N75, L50, and other contiguity characteristics did not change during polishing (Figure 2). The reference-based QUAST statistics, such as genomic features and covered genome fraction, which also describe the assembly completeness, increased after polishing. Indels per 100 kbp and mismatches per 100 kbp improved from 182.4 to 169.0 and from 4425.1 to 4373.7, respectively (Supplementary Table S2). It is not the absolute values of these parameters that play a key role during polishing, but the general trend in improvement.

**Figure 3:**
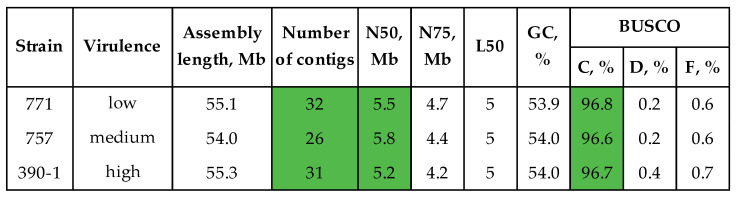
QUAST and BUSCO statistics of the final genome assemblies of *C. lini* strains #390-1, #757, and #771. BUSCO: C—complete, D—duplicated, F—fragmented (the glomerellales_odb10 dataset). Green color is used to highlight the most important statistics.

The algorithm applied for strain #390-1 was used to assemble and polish the genomes of the moderately virulent strain #757 and the lowly virulent strain #771 (assembling with Canu and polishing with Racon ×2–Medaka–Polca). The final assemblies were 54.0–55.3 Mb in length, consisted of 26–32 contigs, and had an N50 of 5.2–5.8 Mb and an L50 of 5 (Figure 3, Supplementary Table S2). The BUSCO completeness of the final assemblies was 96.6–96.8%.

### 3.2. Genome Annotation and Search for Effector Proteins

**Figure 4:**
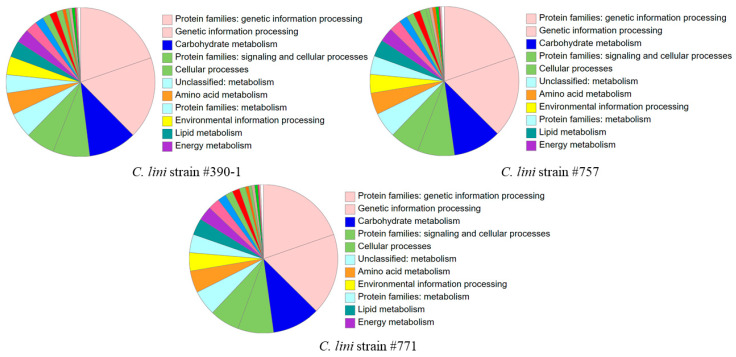
KEGG BlastKOALA statistics of the annotated proteins for the *C. lini* highly virulent strain #390-1, moderately virulent strain #757, and lowly virulent strain #771.

To predict genetic features in the obtained assemblies, we used Funannotate, which was tailored to annotate fungal genomes (Supplementary File S1). Before annotation, the final assemblies were preprocessed by filtering out the repetitive contigs, sorting the input contigs by their size (from the longest to the shortest), relabeling the contigs, and masking repeats. Gene prediction was performed using the basic Funannotate commands. The tool annotated the majority of genes using the Pfam 36.0 (~60% of genes) and BUSCO (~10% of genes) databases. After annotation, we obtained the following: 12,891 gene models (12,521 mRNAs and 370 tRNAs) for the highly virulent strain #390-1, 12,520 gene models (12,146 mRNAs and 374 tRNAs) for the moderately virulent strain #757, and 12,736 gene models (12,374 mRNAs and 362 tRNAs) for the lowly virulent strain #771. All obtained protein sequences were analyzed using KEGG BlastKOALA and InterProScan. In the genome of each strain, KEGG annotated ~33% of proteins and InterPro annotated ~80% of proteins (Supplementary Tables S3 and S4). The largest protein categories in the KEGG annotation were associated with genetic information processing, carbohydrate metabolism, and signaling and cellular processes (Figure 4).

To identify the possible effector proteins, we determined the presence of signal peptides using SignalP and then predicted effectors from the positive hits with EffectorP (Supplementary Table S5). In the highly virulent strain #390-1, 1308 proteins contained a signal peptide, 489 (37.4%) of them were effectors: 187 (14.3%) cytoplasmic effectors and 302 (23.1%) apoplastic effectors. In the moderately virulent strain #757, 1303 proteins contained a signal peptide, 472 (36.2%) of them were effectors: 184 (14.1%) cytoplasmic effectors and 288 (22.1%) apoplastic effectors. In the lowly pathogenic strain #771, 1288 proteins contained a signal peptide, 476 (37.0%) of them were effectors: 191 (14.8%) cytoplasmic effectors and 285 (22.1%) apoplastic effectors. The predicted effectors were also analyzed with KEGG BlastKOALA and InterProScan. We searched for unique accessions in the annotations of the effector proteins. The protein was regarded as unique for a strain if it did not occur in the database annotation of the other two strains. In the KEGG BlastKOALA annotations, unique accessions were found only in the highly virulent strain #390-1 (C_lini_390-1_FUN_004740-T1, C_lini_390-1_FUN_004122-T1, C_lini_390-1_FUN_001342-T1). However, in the InterProScan annotations of effectors, eight accessions were unique for the same strain (three of them were annotated with KEGG) (C_lini_390-1_FUN_004637-T1, C_lini_390-1_FUN_004750-T1, C_lini_390-1_FUN_004740-T1, C_lini_390-1_FUN_004688-T1, C_lini_390-1_FUN_001551-T1, C_lini_390-1_FUN_004122-T1, C_lini_390-1_FUN_001342-T1, C_lini_390-1_FUN_009633-T1), seven accessions in the moderately virulent strain #757 (C_lini_757_FUN_004039-T1, C_lini_757_FUN_005381-T1, C_lini_757_FUN_010924-T1, C_lini_757_FUN_000684-T1, C_lini_757_FUN_003376-T1, C_lini_757_FUN_000430-T1, C_lini_757_FUN_000370-T1), and two accessions in the lowly virulent strain #771 (C_lini_771_FUN_009418-T1, C_lini_771_FUN_009066-T1) (Supplementary Table S6). Unique accessions were related to the metabolism (catabolism) of carbohydrates and nitrogen and cell signaling pathways. This fact points to the possible role of these enzymes in the processes of plant colonization and nutrition, i.e., infection, and suggests the direction of further studies. However, other proteins related to virulence can also exist. To establish the factors of strain differentiation by virulence, further research is needed.

### 3.3. Comparative Genomic Analysis

In each assembly, we identified a mitochondrial contig by blasting the previously obtained sequence of the mitochondrial genome of *C. lini* #811 (highly virulent) [25]. The extracted mitochondrial genomes were aligned to each other in Mega. The mitochondrial genomes had similar sizes: 38,956–39,090 bp. Only several mismatches were observed in the multiple alignment. Furthermore, the annotations of the received mitochondrial genomes had no differences.

**Figure 5:**
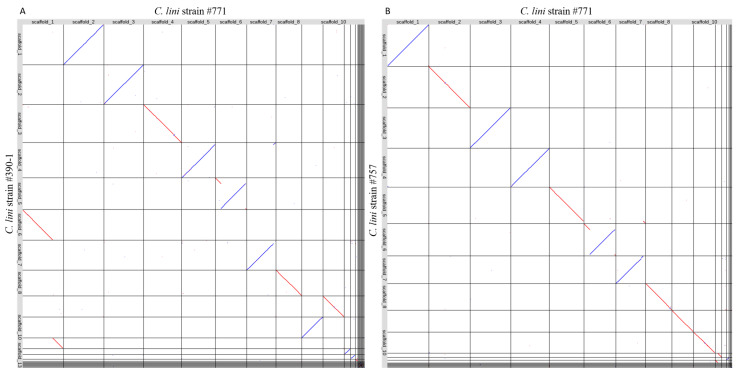
The results of (**A**) the alignment of the genome assemblies of the lowly virulent strain #771 and the highly virulent strain #390-1; (**B**) the alignment of the genome assemblies of the lowly virulent strain #771 and the moderately virulent strain #757. Red lines indicate the forward orientation of the aligned sequences, and blue lines indicate the reverse orientation of the aligned sequences.

The final genome assemblies of three *C. lini* strains were aligned to each other using LAST (Figure 5). The obtained plots showed that the majority of scaffolds of the moderately virulent strain #757 aligned to the scaffolds of the lowly virulent strain #771. However, the scaffolds of the highly virulent strain #390-1 were shifted by one. Scaffold 1 of the strain #771 consisted of scaffolds 6 and 11 of strain #390-1. In scaffold 6 in the lowly virulent strain #771, we detected a rearrangement. The beginning of scaffold 6 and a little part at the end of this scaffold aligned to scaffold 5 in strain #390-1 and scaffold 6 in strain #757 in forward orientation. Meanwhile, the middle part of scaffold 6 aligned in reverse orientation to the same scaffolds in the two other strains. Scaffold 12 in the lowly virulent strain #771 corresponded to scaffold 13 in the highly virulent strain #390-1. Yet a similar scaffold was missing from the genome of the moderately virulent strain #757. To confirm the absence of the missing scaffold, its sequence was blasted to the genome of the strain #757 genome assembly. However, no significant hits were found. The missing scaffold was 0.7 Mb in size. We blasted amino acid sequences of predicted proteins from this scaffold against the NCBI database. The found protein hits were helicases.

Next, we searched for telomeric repeats (‘TTAGGG’) in the obtained genome assemblies using Tidk [46]. The output Tidk files with diagrams of the occurrence frequency of the target sequence are presented in Supplementary Figures S1–S3. High peaks at both ends of a scaffold indicated the presence of telomeric repeats. Such a scaffold could be a complete chromosome. Therefore, the assembled genomes can have from four to six complete chromosomes. We also observed several scaffolds with telomeric repeats at only one end. Assuming the karyotype of n = 13 [47], nearly a third or a half of the assembled contigs could be complete chromosomes. The scaffold missing in the assembly of the moderately pathogenic strain #757 (scaffold 12 in the assembly of the lowly virulent strain #771 and scaffold 13 in the assembly of the highly virulent strain #390-1) had telomeric repeats at both ends. Thus, this genomic locus is likely a complete 0.7 Mb-long chromosome and might be a minichromosome associated with pathogenicity [23].

## 4. Discussion

*Colletotrichum* species are widely distributed plant pathogens which cause significant economic losses. The representatives of the genus are actively studied, including at the level of complete genomes. At the time of writing the manuscript, 270 assemblies of *Colletotrichum* species were deposited in the NCBI Genome database (the size of the genomes is about 50–60 Mb). The advances in long-read sequencing technologies allowed obtaining high-quality genome assemblies of *Colletotrichum* species [48,49]. Thus, high-quality genomes became the basis for further molecular genetic studies. Genomics and transcriptomics of *Colletotrichum* species provided valuable information on the genes regulating their life cycle and the ability to produce proteins and secondary metabolites damaging plant cells [50,51,52,53,54,55,56]. To identify molecular genetic factors that determine pathogenicity, special attention is paid to research on the interaction of *Colletotrichum* species and their hosts [57,58,59]. Using high-quality genome assemblies of *Colletotrichum* species, a range of pathogenicity-associated genome regions were identified, including rapidly evolving regions in telomeres, repeat-rich minichromosomes, clusters of effector genes, and a number of genes co-expressing upon infection of a host [46,60,61,62].

The causative agent of flax anthracnose, *C. lini* (syn. *C. linicola*), has been unfairly deprived of attention in molecular genetic studies. The species was mainly studied using DNA markers [20,63,64]. In this study, we sequenced the genomes of three *C. lini* strains with different virulence on flax and conducted a comparative analysis of the obtained fungal genomes to reveal pathogenicity-associated factors. To exclude the contribution of multiple factors in further genomic analysis, we studied the strains with close morphological characteristics. The strains represented three degrees of virulence—low, medium, and high.

The combination of long ONT reads and short precision Illumina reads allows obtaining high-quality genomes of the fungal pathogens [24,25,65]. In this study, we obtained from ~100× to 160× genome coverage with ONT reads (average read Q ≥ 10), having an N50 from ~5 to 12 kb. Coverage with Illumina data ranged from ~50× to 140×. To construct the most contiguous and complete assemblies, two approaches were tested on the highly virulent strain #390-1. The first was based on constructing a draft assembly from long reads and polishing it with both long and short precision reads. The second approach implied the use of hybrid assembly software, taking both ONT and Illumina data as input. We used recently developed tools and software that demonstrated optimal results in our previous studies [24,25,65]. The most contiguous assembly was obtained using Canu—31 contigs, N50 = 5.2 Mb, L50 = 5. However, its BUSCO completeness (92.1%) was lower than the completeness of the assemblies obtained with hybrid tools. Thus, Masurca and Unicycler assembled genomes with a BUSCO completeness of 95.9%. Since polishing can increase the parameter, the draft assembly by Canu was considered optimal. According to the scheme that showed the best results in our previous studies, the chosen draft assembly was polished using Racon ×2–Medaka (ONT reads) and Polca (Illumina reads) [24,25,65]. Thus, the BUSCO completeness of the assembly rose from 92.1 to 96.7%. The final value was higher than that achieved by Masurca and Unicycler. Thus, the Canu–Racon ×2–Medaka–Polca scheme allowed us to assemble a contiguous and complete genome. The scheme was employed to assemble the genomes of strains #757 and #771. The final genomes consisted of 26–32 contigs, had N50 values in the megabase range (5.2–5.8 Mb), and were more than 96% complete.

Thus, the obtained genomes had high contiguity. After the search for telomeric repeats and their visualization (Supplementary Figures S1–S3), we observed peaks at one or both ends of the obtained contigs. This indicated that the assembled contigs were possibly big parts or complete chromosomes. At the time of writing the manuscript, two chromosome-level assemblies were available in the NCBI database (*Colletotrichum higginsianum* IMI 349063 GCA_001672515.1 and *Colletotrichum graminicola* GCA_029226625.1). The contig N50 values of these two assemblies are 5.2 and 5.0 Mb, respectively. The L50 values for both assemblies are 5. In this study, we constructed contig-level assemblies of *C. lini*. However, the analysis of telomeric repeats suggested the presence of complete chromosomes. Thus, high coverage with long ONT reads probably allowed assembling the sequences of complete chromosomes. Furthermore, the contiguity of the obtained assemblies is comparable to that of the chromosome-level assemblies prior to anchoring to chromosomes.

The assemblies were annotated using Funannotate. The resulting annotations had close numbers of predicted gene models. The highly virulent strain had the highest number of gene models (#390-1)—12,891, and the moderately virulent strain (#757) had the lowest number of gene models—12,520. Meanwhile, strain #757 had lower BUSCO completeness than strain #390-1. Although the completeness of an assembly impacts the accuracy of gene prediction, the highest number of gene models in the genome of the highly virulent strain can still correlate with its high pathogenicity. In *C. graminicola*, ~15,000 genes were predicted [66]. Thus, the number of predicted gene models for *C. lini* was in the order of the values from the literature data. To conduct a primary analysis of virulence genes, we searched for the encoded effector proteins in the obtained genome assemblies. Effector proteins are the small cysteine-rich proteins influencing plant cellular processes to facilitate the infection process [67]. The lowly virulent strain #771 contained the lowest number of proteins with signal sequences, i.e., potentially secreting, and 37.0% of them were predicted as effectors. The highly virulent strain #390-1 had the highest number of potentially secreting proteins and 37.4% of them were predicted as effectors. Meanwhile, the moderately virulent strain #757 had the lowest number of effectors (36.2% of proteins with signal sequences), but it also had the lowest number of gene models. According to protein annotations with KEGG, the genome of the highly virulent strain #390-1 contained the highest number of effectors with unique annotations, and the lowly virulent strain #771 contained the smallest number of effectors with unique annotations. This fact can correlate with the degree of pathogenicity of a strain. In a similar study, the strain of *C. scovillei* was characterized by defective growth and virulence, along with a reduced number of effectors [68]. Possibly, higher numbers of effector proteins and uniquely annotated effectors are related to higher pathogenicity. However, the obtained results can still be prone to fluctuations in predicted values. Furthermore, the detection of an effector protein can also trigger plant immunity mechanisms, decreasing the virulence of a fungus [69]. Therefore, further research is needed to collect more information on the effector proteins of the studied fungi and elucidate true virulence mechanisms.

To reveal the possible effect of genome rearrangements, we performed whole-genome alignment of the three *C. lini* genomes with each other. In the assembly of the lowly virulent strain #771, scaffold 6 contained one big inversion. Such genome rearrangements might be crucial for the function of certain genomic regions. Small scaffold 12 (0.7 Mb) in the lowly virulent strain #771 aligned to scaffold 13 in the highly virulent strain #390-1. However, this sequence was completely missing in the genome of the moderately virulent strain #757. Since this scaffold had an increased occurrence of repeats at its ends, we assumed that it could be a small pathogenicity-associated chromosome [23]. Furthermore, BLAST analysis of the annotated proteins from the scaffold showed that it contained helicases, peptidases, and hydrolases. Therefore, the minichromosome can be implicated in replication events, the growth of the fungus, and necrotrophy.

In this work, using ONT and Illumina data, we obtained the first three high-quality *C. lini* genomes. We performed primary comparative analysis of the obtained assemblies. The difference in the number of effector proteins and the presence of a putative minichromosome suggested possible determinants of the high virulence. The assembled whole genome sequences created the foundation for a further in-depth search for molecular determinants of pathogenicity both at the chromosome and gene levels. Such data are indispensable for the advancement of anthracnose management techniques and conceiving of new strategies for breeding resistant varieties. Moreover, the obtained high-quality genomes of *C. lini* expand the knowledge of the genetic diversity of the genus *Colletotrichum*.

## Data Availability

The generated dataset for this study can be found in the NCBI database under the BioProject accession number PRJNA929545.

## Supplementary Materials

The following supporting information can be downloaded at: https://www.mdpi.com/article/10.3390/jof10010032/s1, Table S1: QUAST statistics of *Colletotrichum lini* strain #390-1 draft genome assemblies; Table S2: QUAST statistics of polished genome assemblies of *Colletotrichum lini* highly virulent strain #390-1, moderately virulent strain #757, and lowly virulent strain #771; Table S3: The full list of InterPro-annotated proteins with IP, definition, and GO terms for *Colletotrichum lini* highly virulent strain #390-1, moderately virulent strain #757, and lowly virulent strain #771; Table S4: The full list of KEGG-annotated proteins of *Colletotrichum lini* highly virulent strain #390-1, moderately virulent strain #757, and lowly virulent strain #771; Table S5: The full list of effector proteins predicted by EffectorP for *Colletotrichum lini* highly virulent strain #390-1, moderately virulent strain #757, and lowly virulent strain #771; Table S6: The list of InterPro annotations for unique effector proteins predicted by EffectorP for *Colletotrichum lini* highly virulent strain #390-1, moderately virulent strain #757, and lowly virulent strain #771; Figure S1: Telomeric repeat (TTAGGG) content along the contig sequences of *Colletotrichum lini* strain #390-1; Figure S2: Telomeric repeat (TTAGGG) content along the contig sequences of *Colletotrichum lini* strain #757; Figure S3: Telomeric repeat (TTAGGG) content along the contig sequences of *Colletotrichum lini* strain #771; File S1: Genome annotations for three *Colletotrichum lini* strains of high (#390-1), medium (#757), and low (#771) virulence in gbk and gff3 formats.
