# Supplementary material for: Comparative Genomic Analysis of Colletotrichum lini Strains with Different Virulence on Flax
Source: J Fungi (Basel). 2023 Dec 31;10(1):32. doi: 10.3390/jof10010032 (PMC10817032; doi:10.3390/jof10010032)
Supplement: Supplementary file 1 [file jof-10-00032-s001.zip › Supplementary Figure S1_2023.12.03.pdf]

Figure S1. Telomeric repeat (TTAGGG) content along the contig sequences of *Colletotrichum lini* strain #390-1: horizontal axis - coordinate, vertical axis - repeat occurence (frequency)

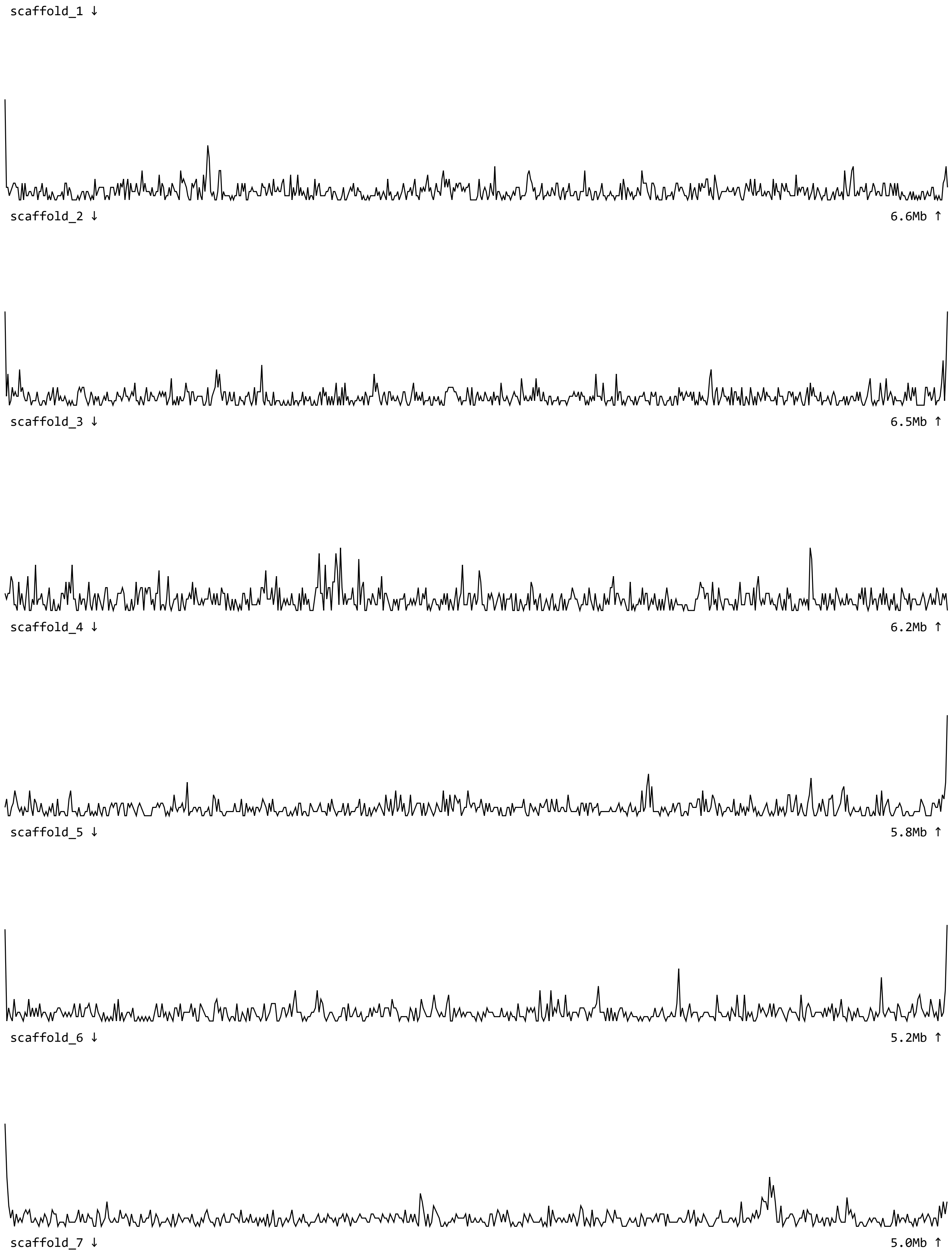

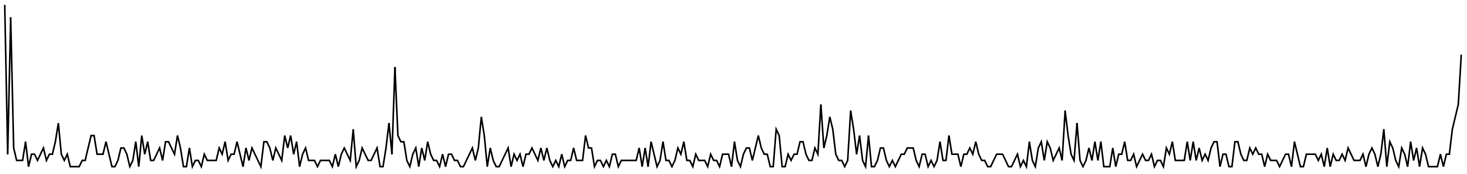
scaffold_8 ↓
4.9Mb ↑

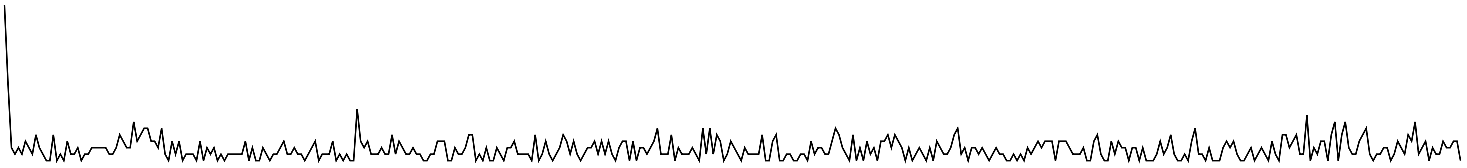
scaffold_9 ↓
4.2Mb ↑

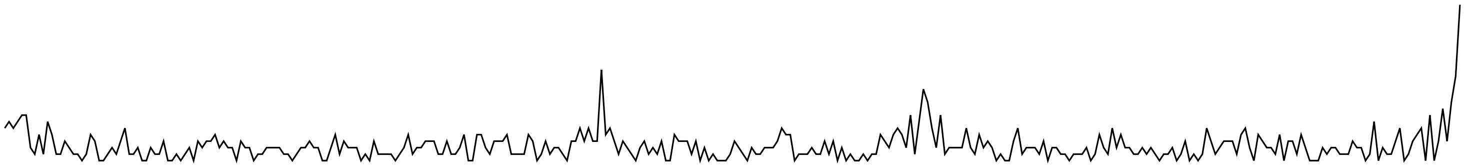
scaffold_10 ↓
3.4Mb ↑

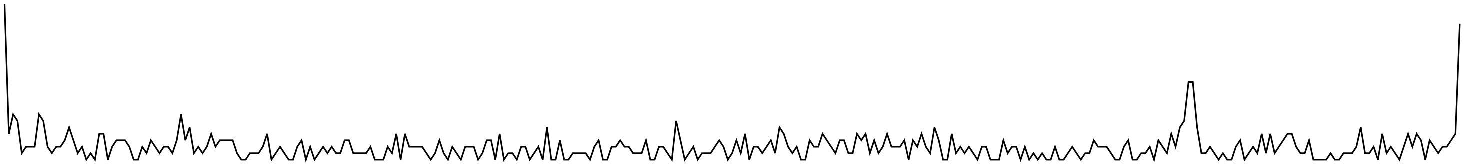
scaffold_11 ↓
3.4Mb ↑

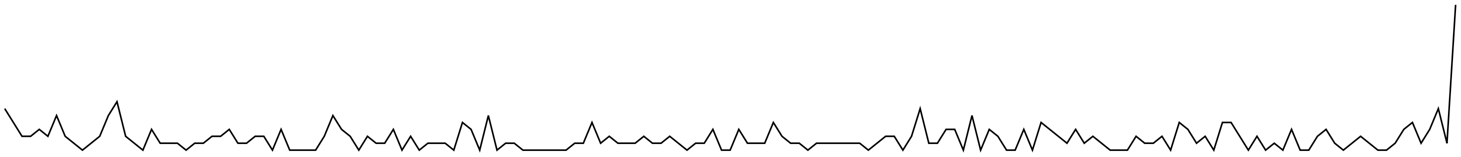
scaffold_12 ↓
1.7Mb ↑

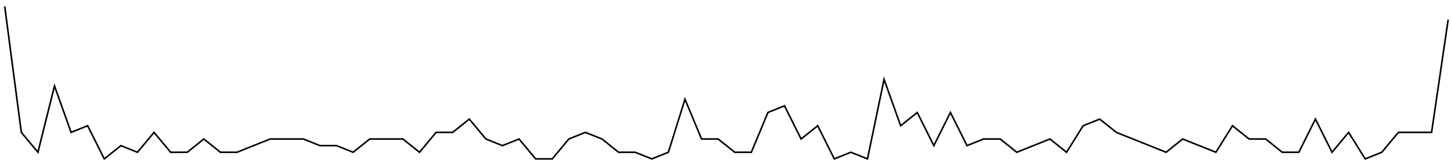
scaffold_13 ↓
0.9Mb ↑

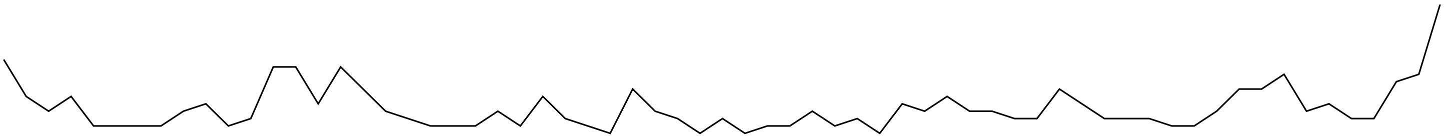

scaffold_14 ↓ 0.7Mb ↑

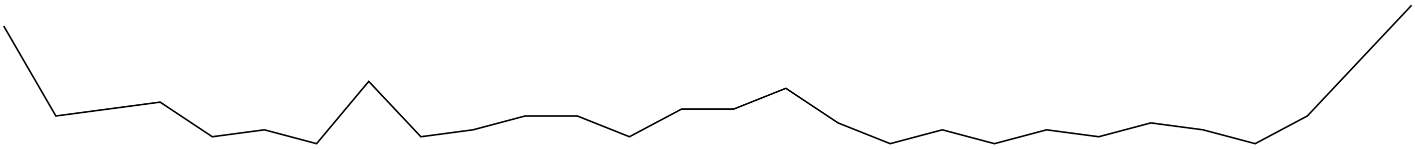

scaffold_15 ↓ 0.3Mb ↑

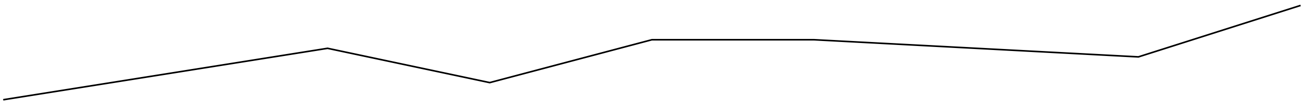

scaffold_16 ↓ 0.1Mb ↑

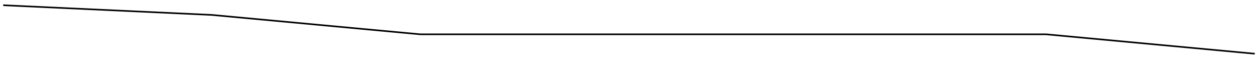

scaffold_17 ↓ 0.1Mb ↑

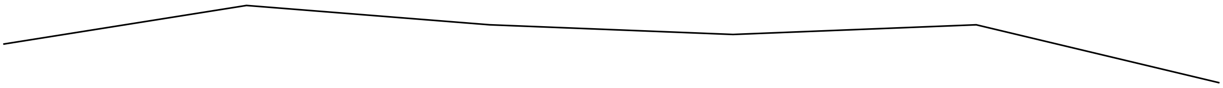

scaffold_18 ↓ 0.1Mb ↑

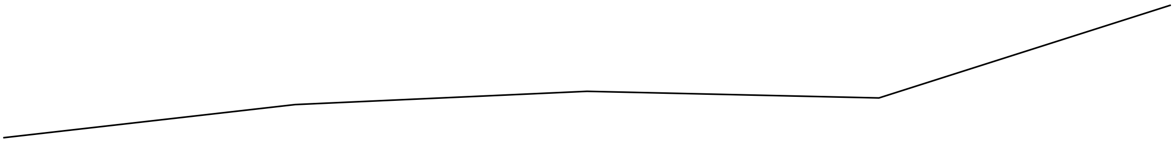

scaffold_19 ↓ 0.1Mb ↑

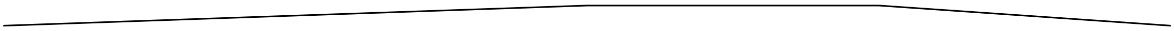

scaffold_20 ↓ 0.1Mb ↑

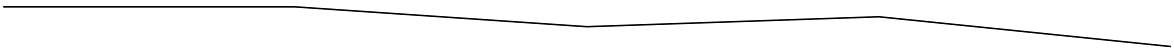

scaffold_21 ↓ 0.1Mb ↑

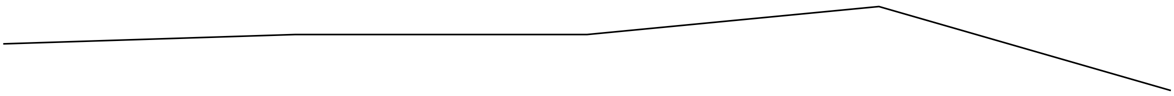

scaffold_22 ↓ 0.1Mb ↑

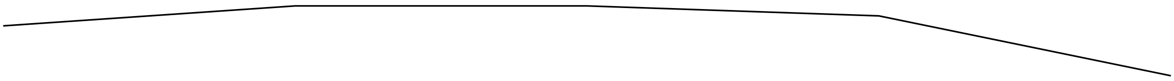

scaffold_23 ↓ 0.1Mb ↑

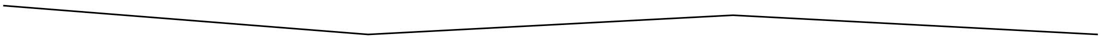

scaffold_24 ↓ 0.0Mb ↑

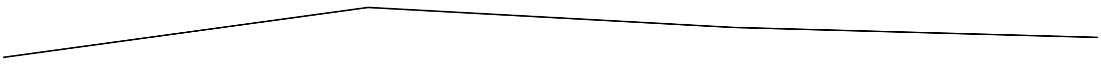

scaffold_25 ↓ 0.0Mb ↑

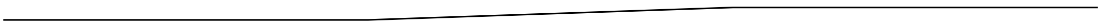

scaffold_26 ↓ 0.0Mb ↑

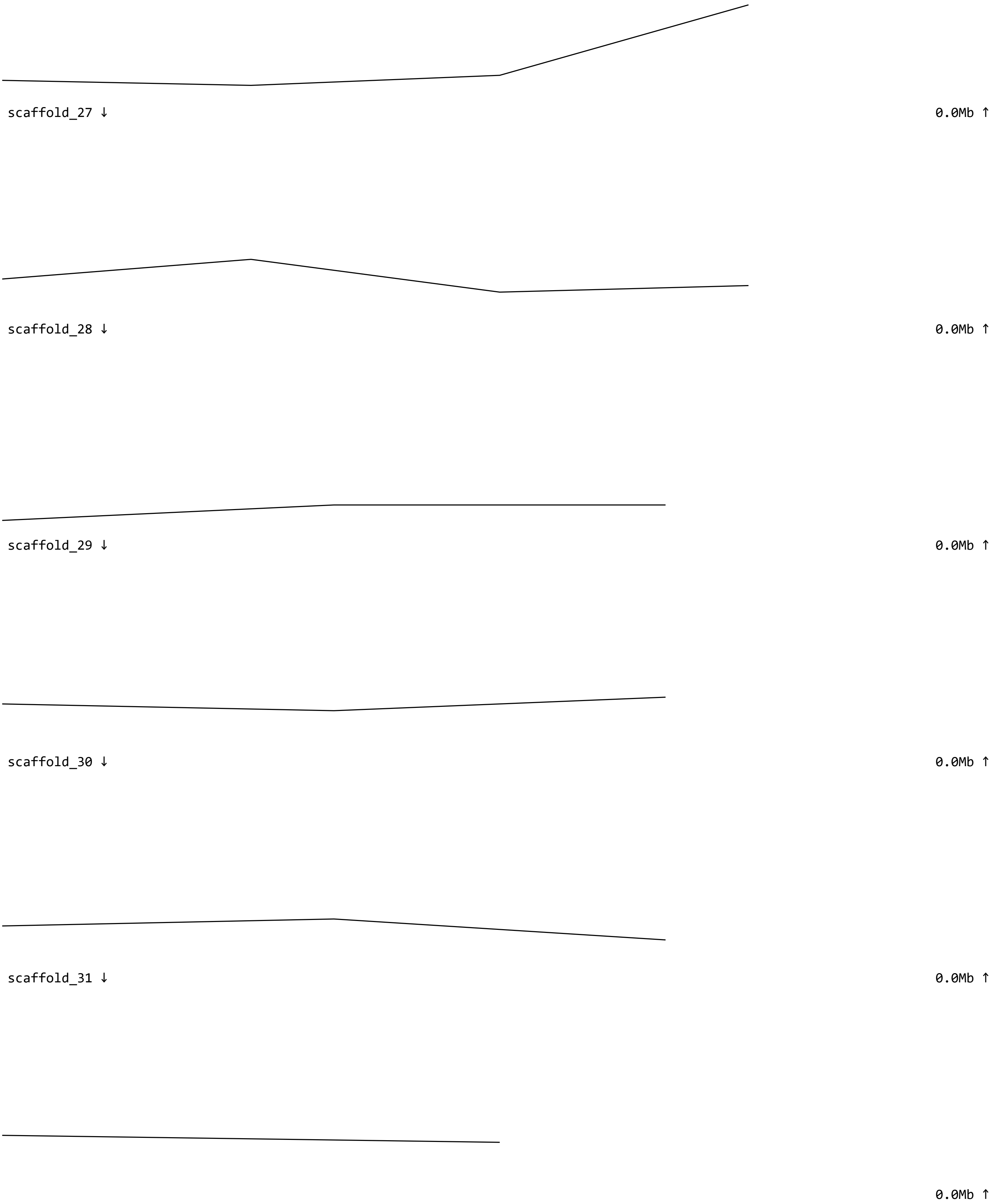
scaffold_27 ↓
0.0Mb ↑
scaffold_28 ↓
0.0Mb ↑
scaffold_29 ↓
0.0Mb ↑
scaffold_30 ↓
0.0Mb ↑
scaffold_31 ↓
0.0Mb ↑
0.0Mb ↑
