# Supplementary material for: Comparative Genomic Analysis of Colletotrichum lini Strains with Different Virulence on Flax
Source: J Fungi (Basel). 2023 Dec 31;10(1):32. doi: 10.3390/jof10010032 (PMC10817032; doi:10.3390/jof10010032)
Supplement: Supplementary file 1 [file jof-10-00032-s001.zip › Supplementary Figure S3_2023.12.03.pdf]

Figure S3. Telomeric repeat (TTAGGG) content along the contig sequences of *Colletotrichum lini* strain #771: horizontal axis - coordinate, vertical axis - repeat occurence (frequency)

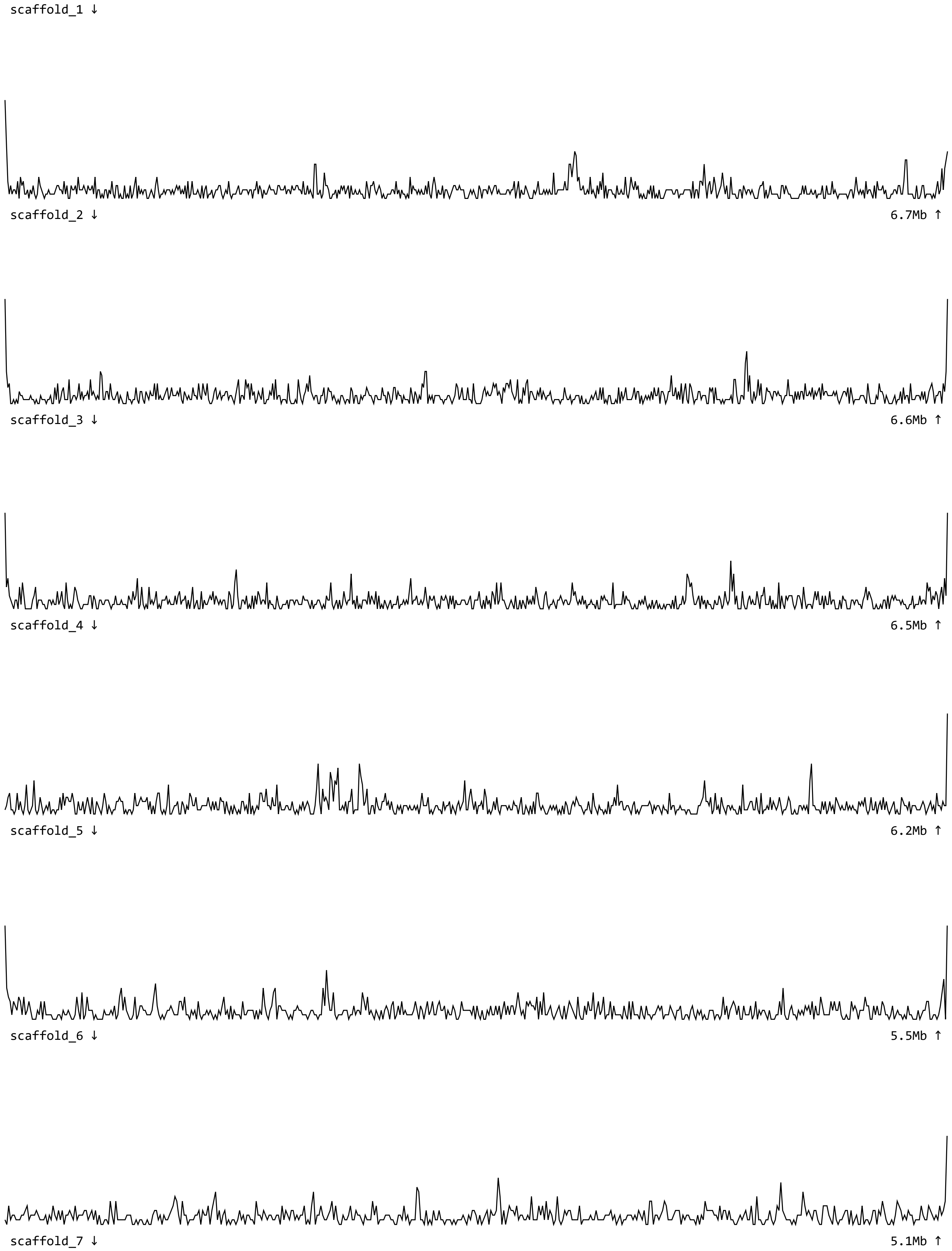

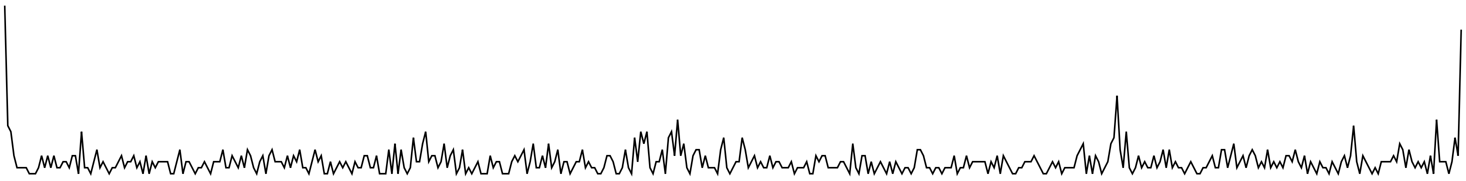

scaffold_8 ↓ 4.8Mb ↑

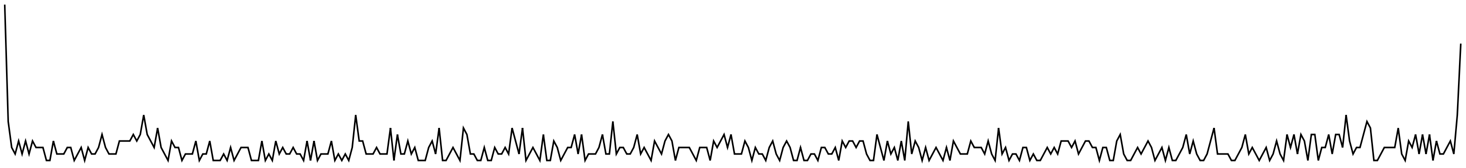

scaffold_9 ↓ 4.2Mb ↑

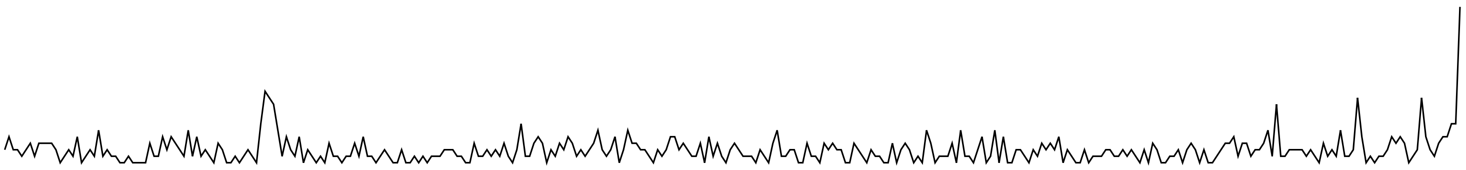

scaffold_10 ↓ 3.4Mb ↑

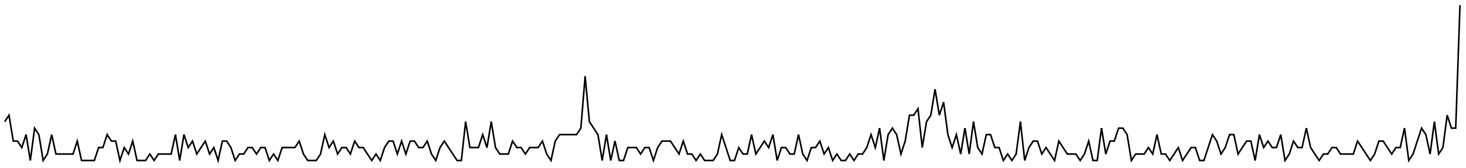

scaffold_11 ↓ 3.4Mb ↑

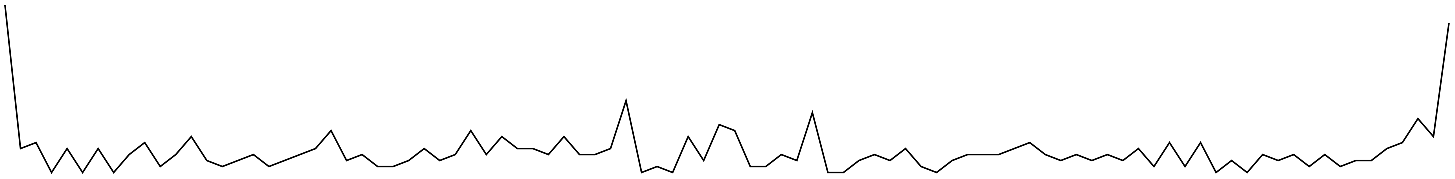

scaffold_12 ↓ 0.9Mb ↑

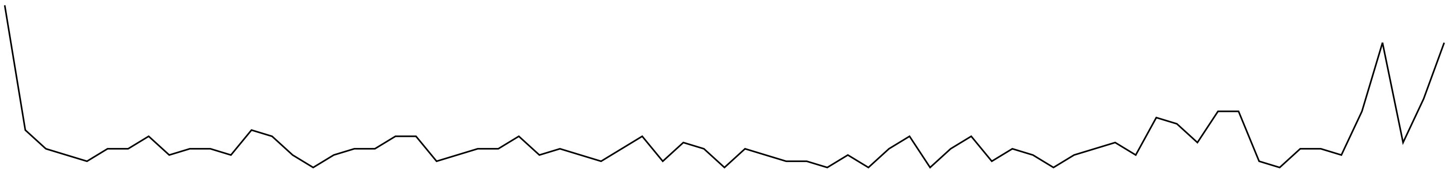

scaffold_13 ↓ 0.7Mb ↑

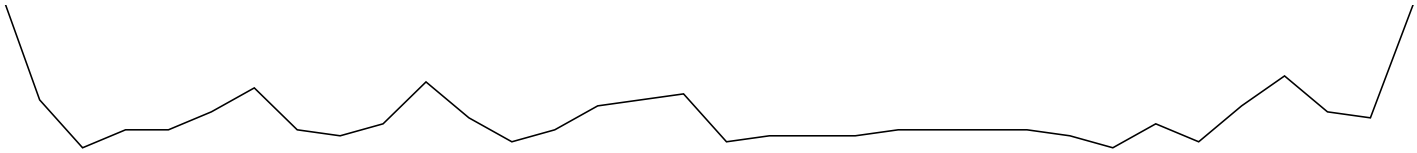

scaffold_14 ↓ 0.3Mb ↑

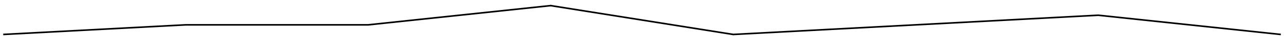

scaffold_15 ↓ 0.1Mb ↑

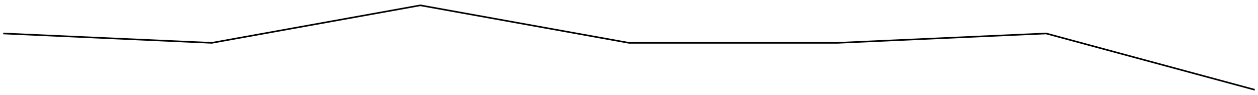

scaffold_16 ↓ 0.1Mb ↑

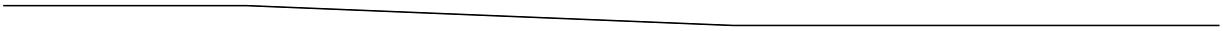

scaffold_17 ↓ 0.1Mb ↑

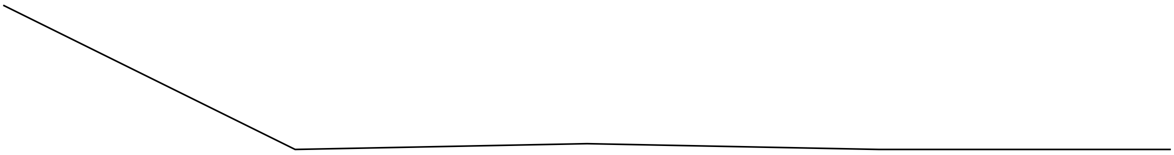

scaffold_18 ↓ 0.1Mb ↑

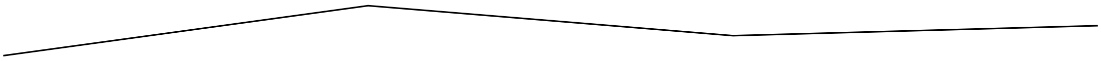

scaffold_19 ↓ 0.0Mb ↑

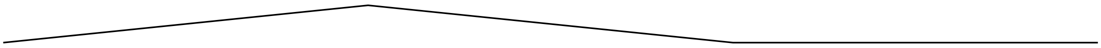

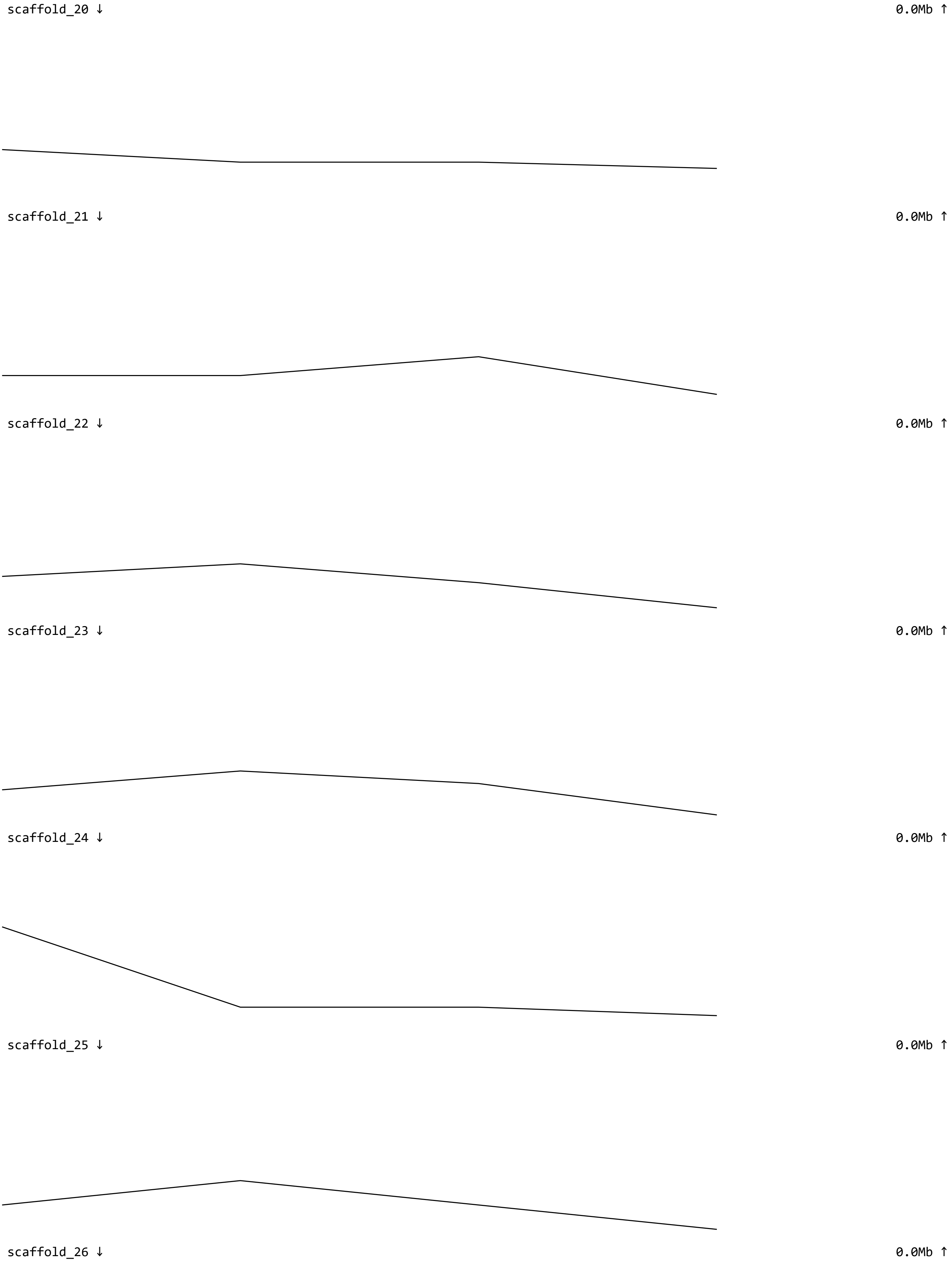
scaffold_20 ↓
0.0Mb ↑
scaffold_21 ↓
0.0Mb ↑
scaffold_22 ↓
0.0Mb ↑
scaffold_23 ↓
0.0Mb ↑
scaffold_24 ↓
0.0Mb ↑
scaffold_25 ↓
0.0Mb ↑
scaffold_26 ↓
0.0Mb ↑

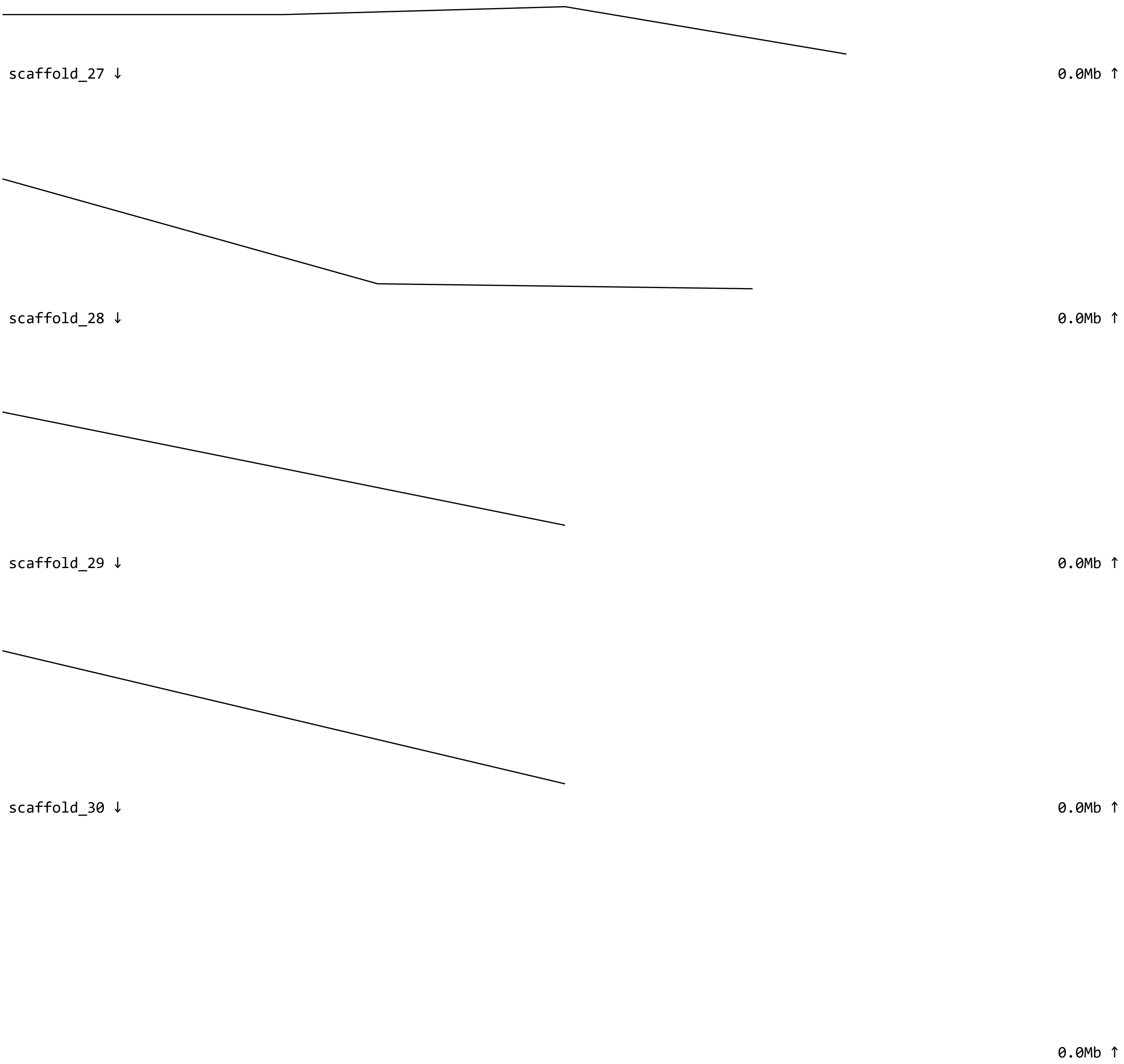
scaffold_27 ↓
0.0Mb ↑
scaffold_28 ↓
0.0Mb ↑
scaffold_29 ↓
0.0Mb ↑
scaffold_30 ↓
0.0Mb ↑
0.0Mb ↑
