# Supplementary material for: Comparative Genomic Analysis of Colletotrichum lini Strains with Different Virulence on Flax
Source: J Fungi (Basel). 2023 Dec 31;10(1):32. doi: 10.3390/jof10010032 (PMC10817032; doi:10.3390/jof10010032)
Supplement: Supplementary file 1 [file jof-10-00032-s001.zip › Supplementary_File_S1.pdf]

Supplementary File S1. Genome annotations for three *Colletotrichum lini* strains of high (#390-1), medium (#757), and low (#771) virulence in gbk and gff3 formats.

Archive content:
1) Supplementary_File_S1 (description),
2) C_lini_390-1.gbk,
3) C_lini_390-1.gff3,
4) C_lini_757.gbk,
5) C_lini_757.gff3,
6) C_lini_771.gbk,
7) C_lini_771.gff3.

The archive contains genome annotation files for three *Colletotrichum lini* strains and a description.
GBK files contain DNA sequences in a plain text format and additional data such as sample source, and description.
GFF3 files contain information on genome features (location, strand location, feature name).
To open the attached files, you can use a text-editing tool installed on your system, e.g., Windows Notepad.
Right click on the file, choose "open with ...", choose your text-editing tool.
